# Co-existing sarcoidosis and Takayasu arteritis: report of a case

**DOI:** 10.1186/1755-7682-4-9

**Published:** 2011-02-23

**Authors:** Amira Hamzaoui, Randa Salem, Rim Klii, Olfa Harzallah, Olfa Berriche, Mondher Golli, Silvia Mahjoub

**Affiliations:** 1 Department of Internal Medicine- Fattouma Bourguiba Hospital- Monastir- Tunisia; 2 Department of Radiology- Fattouma Bourguiba Hospital- Monastir- Tunisia

## Abstract

**Introduction:**

Takayasu arteritis (TA) is a chronic vasculitis of unknown origin, affecting mainly the aorta and its main branches. As a result of the inflammation, stenosis, occlusion or dilatation of the involved vessels may occur and cause a wide range of symptoms. It has been described in association with various auto-immune disorders (mainly inflammatory digestive tract diseases). However, only few cases of TA associated with sarcoidosis have been reported, raising the question of an association by chance.

**Case report:**

We report a case of a 34 year-old woman, with one year history of sarcoidosis, who presented with asymmetric high hypertension revealing inflammatory humeral, axillary and subclavian arteritis related to TA, successfully treated by steroid and immunosuppressive therapy(Methotrexate^R^).

**Conclusion:**

TA and sarcoidosis may be related, rising the hypothesis that TA or Takayasu arteritis-like granulomatous vasculitis may be, in fact, a complication of sarcoidosis.

## Introduction

Takayasu arteritis (TA) is a chronic inflammatory disease of large arteries which progressively develop stenosis, occlusion or aneurismal degeneration. The clinical presentation is characterised by an acute phase with constitutional symptoms, followed, months or years later, by a chronic phase in which symptoms relate to fibrosis or occlusion of vessels. Angiography is the gold standard for diagnosis and for topographical classification and it correlates with symptoms and prognosis. Proinflammatory cytokines and, among these, tumor necrosis factor-alpha (TNF-alpha) are increased and play a pathogenetic role in the development of disease. Laboratory findings are non-specific. Treatment of active disease is primarily based on corticosteroids but other immunosuppressive drugs are frequently needed. Anti-platelets agents, statins and antihypertensive drugs are frequently considered [1].

Sarcoidosis, too, is a systemic inflammatory disease which can affect virtually any organ system. The lungs are most commonly affected, but often there is also affection of the musculoskeletal system, the cutaneous and central nervous systems, and the heart. Histology shows granulomas consisting of a central zone with macrophages, epithelioid cells, and multinucleated giant cells in addition to activated CD4 lymphocytes, and a peripheral zone with macrophages, fibroblasts, and CD4 and CD8 lymphocytes. Inclusion bodies are commonly found. Vasculitis may be found occasionally in patients with sarcoidosis. It has not been confirmed, however, whether vasculitis is a true manifestation of the disease or an occasional, incidentally concurring abnormality.

Concurrence of sarcoidosis and TA were reported in few cases.

## Case Report

A 34 year old woman was admitted to the hospital with dyspnea, submandubular gland tumefaction and parotid swelling.

On physical examination, we found the tumefaction of the parotid and the submandibular gland. The respiratory frequency was 24 cycles per minute. The rest of the clinical examination was without abnormalities.

Laboratory results were as follows: Haemoglobin 10 g/dl, white blood cell count (WBC) 11 200/mm3, platelets 450 000/mm3, erythrocyte sedimentation rate (ESR) 76 mm/h, calcium 12 mg/dl (normal: 9-11 mg/dl ). Muscle enzymes, ANA, rheumatoid factor, VDRL, and complement components C3 and C4 were negative. The angiotensin converting enzyme (ACE) level was 135 UI/L.

Chest radiography showed "Bilateral interstitial involvement". Thoracoabdominal CT scan showed "Multiple mediastinal and retroperitoneal lymphadenopathy, with moderate hepatomegaly". The respiratory functional test showed the presence of a restrictive syndrome.

The lung fibroscopy was normal. The biopsy confirmed the presence of a non caseating granuloma in the bronchial mucosa. The bronchoalveolar lavage showed a predominantly lymphocytic cell with the elevated Cd4/CD8 ratio.

Ophthalmological examination confirmed the presence of dry eye syndrome. There was no uveitis. The salivary gland biopsy confirmed the presence of a granuloma without caseous necrosis.

Sarcoidosis was diagnosed, and the patient received prednisolone 60 mg/day. After 1 month of treatment, all complaints have been resolved.

Seven months later, when she received 10 mg/day of prednisolone, she consulted for respiratory distress. Her left brachial and radial pulses were absent, and her blood pressure was 180/120 mm Hg as measured from the left arm. Bruits were audible over the course of the left carotid artery and the left and the right subclavian artery. The patient had elevated ERS (100 mm/H).

**Figure 1:**
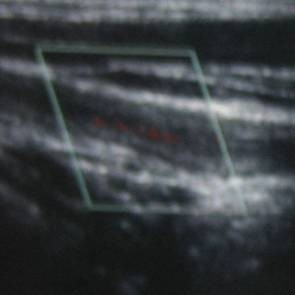
USCD of the right carotid artery showing circumferential parietal swelling interesting the left subclavian artery reducing his superficies of 76% and demodulating the Doppler spectrum

An Ultra Sound Colour Doppler (USCD) was performed showing a circumferential parietal swelling interesting the left subclavian artery reducing his superficies of 76%, demodulating the Doppler spectrum and edema of the vessl wall[Figure 1]. These data were confirmed by MR-agniography.

We diagnosed the patient having TA associated with sarcoidosis. She received prednisolone 40 mg/day with Methotrexate 10 mg/week and amlodipine 5 mg/say.

As a response to treatment, blood pressure was normal 2 weeks after. The USCD control confirmed the therapeutic benefice showing a reduction of the parietal anomalies.

## Discussion

About a dozen cases of the association TA and sarcoidosis were reported in the literature. [1-6]; some of them are summarized in table 1. When reviewing 60 patients with TA, Kerr and all found two cases of sarcoidosis associated with TA (3%) [2]. Weiler and all [1], noted that the diagnosis of sarcoidosis has generally preceded (80% cases) TA (2 to 17 years). The discovery of two contemporary conditions is less usual. Our observation corroborates these results, since sarcoidosis preceded by 7 months the onset of TA.

**Table 1:** Features of related cases with coincidence of sarcoidosis and Takayasu arteritis

Author	Sex/Age (years)	Chronology	Clinical complaint	Laboratory results	USCD/CT scan	Treatment
**Weiler **[1]	Female/39	12 year history of sarcoidosis	Cardiac failure transient ischaemic attack	ESR 30 mm/h	-	Surgery Glucocorticoid

**Kerr **[2]	Female/32 ans	Concomitant	Polyarthritis, uveitis	-	Subclavian steal syndrome	-

**Korkmaz **[3]	Female/29	4 year history of sarcoidosis	Pain in the left arm, 15 kg weight loss erythema nodosum diminished left radial pulse	Haemoglobin 11 g/dl, WBC 15 700/mm3, ESR 70 mm/h.	Complete obstruction of the left subclavian, left vertebral and superior mesenteric arteries, and diffuse narrowing in the left common carotid artery	Azathioprine 150 mg/day+ Prednisolone 60 mg/day

**Schapiro **[4]	Female/42	2 year history of sarcoidosis	Arteritis claudication, stenocardia	-	Diffuse narrowing of Aortic bifurcation and subclavian arteries	Glucocorticoid, cyclophosphamide

**Taeib **[5]	Female/11	2 year history of sarcoidosis	Headache, hypertension	-	Stenosis of Aortic arch	-

**Robaday **[6]	Female/26	1 year history of sarcoidosis	Right painful upper limb	Haemoglobin 10,4g/dl, ESR 80 mm/h.	Inflammatory humeral, axillary and subclavian arteritis	Presdnisolone 55 mg/day

USCD: Ultra Sound Colour Doppler; WBC: white blood cell count; ESR: erythrocyte sedimentation rate

TA can be associated with Crohn's disease [7], RCH, systemic lupus erythematosus [8], rheumatoid arthritis, Wegener's granulomatosis [1]. In the other hand, coexistence of sarcoidosis with other vasculitis was rarely reported, such as Wegener's granulmomatosis and Horton disease [9].

The association between sarcoidosis and TA or other forms of aortitis may be rare. However, TA or similar forms of aortitis are too rare to indicate that the reported concurrence with sarcoidosis is merely governed by chance. In fact, all these patients had several common features: (a) usually sarcoidosis preceded TA; (b) the time between the diagnosis of sarcoidosis and that of TA was several years (eight or more) in most of the patients; (c) the aorta and/or its major branches was affected; (d) 50% of the patients had uveitis, which is much less common in frank sarcoidosis; and (e) all patients responded to glucocorticoid treatment.

Association of TA and sarcoidosis raise a number of questions of a possible link between these 2 diseases. Vascular injury (or "granulomatous vasculitis), and especially the location in the aorta thoracic and/or its branches, could be a complication in the course of sarcoidosis [1-3,10]. However, since an association of TA with other granulomatous vasculitis (Crohn's disease) has been described, other authors have suggested that different pathogenic mechanisms might contribute to the occurrence of this association: Infectious factors (Mycobacterium tuberculosis, Chlamydia pneumoniae), genetic factors or immunological disturbances [11,12].

## Conclusion

The association between sarcoidosis and TA are rare but not accidental; complete vascular clinical examination should be performed in those patients in order to detect asymptomatic underlying inflammatory arteritis.

The relatively low prevalence of sarcoidosis, and even more so the rarity of aortitis, suggests that this number is relatively high and too high to be a coincidence; can we say that sarcoidosis and TA appears to be 2 related diseases or that Takayasu arteritis or Takayasu arteritis-like granulomatous vasculitis may be, in fact, a complication of sarcoidosis?

## Consent

Informed consent was obtained from the patient for publication of this case report and accompanying images. A copy of the written consent is available for review by the Editor-in-Chief of this journal.

## Competing interests

The authors declare that they have no competing interests.

## Authors' contributions

AH, RK, OH, OB and SM: Diagnosis the disease and monitoring the patient RS and MG: Have carried out the radiological explorations.

All authors read and approved the final manuscript.
